# Comparison of the placebo effect between different non-penetrating acupuncture devices and real acupuncture in healthy subjects: a randomized clinical trial

**DOI:** 10.1186/s12906-016-1477-2

**Published:** 2016-12-15

**Authors:** Leonardo Yung dos Santos Maciel, Paula Michele dos Santos Leite, Mauricio Lima Poderoso Neto, Andreza Carvalho Rabelo Mendonça, Carla Carolina Alves de Araujo, Jersica da Hora Santos Souza, Josimari Melo DeSantana

**Affiliations:** 1 Post Graduate Program in Health Sciences, Federal University of Sergipe, Rua Cláudio Batista, s/n, Santo Antônio, 49060-100 Aracaju, SE Brasil; 2 Department of Physical Therapy, Federal University of Sergipe, Rua Cláudio Batista, s/n. Bairro Santo Antônio, CEP 49060-100 Aracaju, Sergipe Brasil; 3 Professor of the Department of Physical Therapy and Post Graduate Programs in Health Sciences and Physiological Sciences, Federal University of Sergipe, Rua Cláudio Batista, s/n. Bairro Santo Antônio, CEP 49060-100 Aracaju, Sergipe Brasil

**Keywords:** Acupuncture, Placebo, Acupuncture Points, Healthy Volunteers

## Abstract

**Background:**

Several studies have used placebo acupuncture methods in recent years as a way for blinding therapeutic effect of acupuncture, however placebo method selection has not followed enough methodological criteria to the point of stabilishing a consensus of what should be the best method to be used. This study aimed to evaluate the effectiveness of three different placebo acupuncture methods for blinding applied in healthy subjects.

**Methods:**

This study was approved by the Ethics Committee of the Federal University of Sergipe with the number 47193015.5.0000.5546 and all individuals participating in the study signed a free and informed consent. For this study, 321 healthy volunteers were randomly divided into seven groups using the abdominal point stomach (ST) 25 and seven groups using the lumbar point bladder (Bl) 52 for stimulation. For real acupuncture procedure, three different methods of placebo acupuncture plus a mix between real acupuncture and placebo applied in the same individual, totaling fourteen groups in this study. Outcome assessments were performed before and immediately after applying the technique. Investigator who assessed variables had no knowledgement about the method was applied. Identification, weight and height were measured before puncture by using. At the end, subjects were asked if they believed they were receiving real or placebo acupuncture.

**Results:**

There was no significant difference between groups for the perception about the type o stimulation (wheter real or placebo puncture). Percentage of subjects who reported to have received real acupuncture in the abdominal point was 69.56% in real group, 86.95% in group Park Sham, 82.60% in needle + foam, 91.30% in insertion and removal, 78.26% in real + Park Sham, 86.36% in real + needle and foam, 86.95% in real + insertion and removal, and for the lumbar point was 86.36% in real group, 86.95% in group Park Sham, 69.56% in needle + foam, 72% in insertion and removal, 86.95% in real + Park Sham, 81.81% in real + needle and foam and 78.26% in real + insertion and removal.

**Conclusion:**

All placebo acupuncture methods proposed in this study were equally effective for bliding the study participants using either abdominal or lumbar acupoints, and none of the placebo methods presented benefit compared to the other to be used in future clinical trials.

**Ethics Committee:**

Federal University of Sergipe (UFS), number of approval: 47193015.5.0000.5546

**Trial registration:**

ensaiosclinicos.gov.br RBR-3w2p32 Registered in 28th January 2016.

## Background

In the last decades, acupuncture treatment became popular in the Western world because of its therapeutic effects. However, studies have reported conflicting results when using a control group in research to test the true effectiveness of real acupuncture [1]. Some studies have consistently shown that both real and placebo acupuncture had advantages over untreated control groups [2, 3], while some studies have suggested that real acupuncture is more effective than placebo [4–6]; others have failed to show benefits of real acupuncture over placebo one [7–10].

Although the reasons for such contradictory results are not fully clarified, they call attention to a further investigation about placebo-controlled groups in researches in acupuncture field [1, 11, 12]. Randomized clinical trials double blinded serve as gold standard when comparing the effects of an active against placebo-controlled group. [13]. In clinical trials with acupuncture or any other type of device or drug, the ideal is that the control device used is physiologically inert and indistinguishable from real treatment to not produce therapeutic effects and to provide comparison with real treatment group [1].

Clinical trials that incorporate these controls by using placebo gained notoriety because they tried to follow the concept of control as ideal in literature. Patients must not detect the type of treatment they are receiving [14, 15]. In some cases, the investigator who applies acupuncture also has no knowledgement if the application was real or placebo [16].

The lack of a consensus led to the development and use of various placebo acupuncture devices used in the scientific setting, such as the Streitberger device [17], Park Sham device [16], devices using adhesive foam on the skin to prevent the needle penetration into the skin [1, 18, 19], application with toothpick [20, 21] or pressure at the point only with the guide tube [22]. Some authors develop their own placebo acupuncture strategies, but these are not always visually similar to real acupuncture, which does not allow the application in visible regions of patients [1].

To minimize this gap in the literature, this study selected three distinct placebo acupuncture techniques widely used in clinical trials such as 1) Park sham device, 2) insertion and removal of the needle and 3) needle and foam. Only the Park Sham can be used as a double blinded. Our study aimed to investigate whether the placebo acupuncture techniques are indistinguishable from each other and also to the real acupuncture. Moreover one wanted to identify which placebo device is more effective for bliding the subjects when applied to both abdominal and lumbar points.

## Methods

### Type of Study

This is a randomized, double-blinded, placebo-controlled clinical trial. Random distribution was performed by using sequentially numbered, opaque and sealed envelopes, containing numbers from 1 to 14, corresponding to 14 study groups. Randomization was blocked in a proportion of 1:1, in order to assure proportionality of the number of subjects allocated in 14 groups.

Two types of investigators participated in the study: investigator 1 and investigator 2. Investigator 1 was responsible for the evaluation of subjects and measurement of all variables, before and after treatment. Investigator 2 held the administration of treatment, applying the technique of acupuncture. Investigator 1 didn’t know which treatment patient was receiving. This procedure ensured that the study was double-blinded, since the subjects evaluated were instructed to not look at the needles during the procedure and only investigator 2 had knowledgement of the technique used.

### Sample

Only healthy subjects with no pain or discomfort in the region selected for the puncture, aging 18 years or more who have never received treatment by acupuncture were recruited to this study.

Exclusion criteria included: 1) pregnant or who have recently given birth with birth in the last 3 months; 2) cutaneous lesions in the puncture site; 3) active infectious processes; 4) nerve tissue or disease affecting the region of dermal puncture; 5) inability to understand the instructions or consent to the study; 6) psychiatric disorders; 7) presence of auditory, visual or communication disturbance; or 8) moderate or severe cognitive or psychiatric disorder.

Recruitment of subjects occurred at the buildings of the Federal University of Sergipe, and after acceptation to be included they got through the screening room and were considered fit to participate in the survey, then they were conducted to carry out the initial steps. Only after these measures subjects were taken to the room where the investigator 2 was to indicate in which group the subject would be allocated, and performed the procedure holding the person lying on the stretcher for the same length of time that the accomplished in all groups, to send it back to the room, where all measures were carried out with the investigator 1.

For the calculation of sample size, intensity of discomfort was considered as the main outcome from the pilot study. Assuming standard deviation = 1.7, difference to be detected = 2 (from 0 to 10), significance level = 5%, power test = 95%, obtaining a minimum sample size of 21 subjects in each group.

### Ethical Aspects

This study was approved by the Ethics Committee for Research with Humans at the Federal University of Sergipe (UFS), with CAAE number: 47193015.5.0000.5546 and Report number 1275651. Also, it was recorded in the Brazilian Plataform of Clinical Trials, with registration number RBR-3w2p32. All subjects included in the study signed the informed consent form prior to the evaluation.

### Study Groups

Subjects were randomly allocated into 14 study groups in accordance with the Table 1 below.

**Table 1:** Groups investigated, demographic, anthropometric and distribution of the sample. Values expressed as mean ± standard deviation. Kruskal Wallis, *p* = 0.015

Group	Acupoint	Age	BMI	Sex	Sex	Subject
		(years)	(kg/m^2^)	Fem.(%)	Men.(%)	(n)
Real	St25	24,3 ± 8,8	21,7 ± 2,7	78,25	21,75	23
Park Sham	St25	25,3 ± 5,4	23,8 ± 2,1	52,17	47,83	23
Needle + Foam	St25	26,6 ± 7,8	25,8 ± 3,8	52,17	47,83	23
Insertion and Removal	St25	24,7 ± 7,0	22,5 ± 3,5	86,95	13,05	23
Real + Park Sham	St25	28,3 ± 8,8	24,7 ± 4,7	86,95	13,05	23
Real + Needle and foam	St25	24,3 ± 5,5	23,8 ± 4,2	95,45	4,55	22
Real + insertion and remo.	St25	21,6 ± 3,4	22,9 ± 3,7	82,60	17,4	23
Real	Bl52	24,3 ± 7,1	24,1 ± 6,9	81,81	18,19	22
Park Sham	Bl52	25,1 ± 7,7	23,0 ± 3,9	86,95	13,05	23
Needle + foam	Bl52	29,2 ± 8,0	24,0 ± 5,1	69,56	30,44	23
Insertion and Removal	Bl52	24,6 ± 6,5	22,8 ± 5,7	76	24	25
Real + Park Sham	Bl52	30,8 ± 7,6	23,7 ± 3,1	52,17	47,83	23
Real + Needle and foam	Bl52	27,0 ± 8,3	24,2 ± 4,8	77,27	22,73	22
Real + insertion and remo.	Bl52	25,4 ± 6,7	22,7 ± 3,1	78,26	21,74	23
General:		25,8 ± 7,0	23,5 ± 4,1	75,46	24,54	321
*P* value				*p* = 0.015		

Points selected for this study were BL52 and ST25, with only one of these points investigated in each group and punctures occurred bilaterally in all groups.

**Fig. 1:**
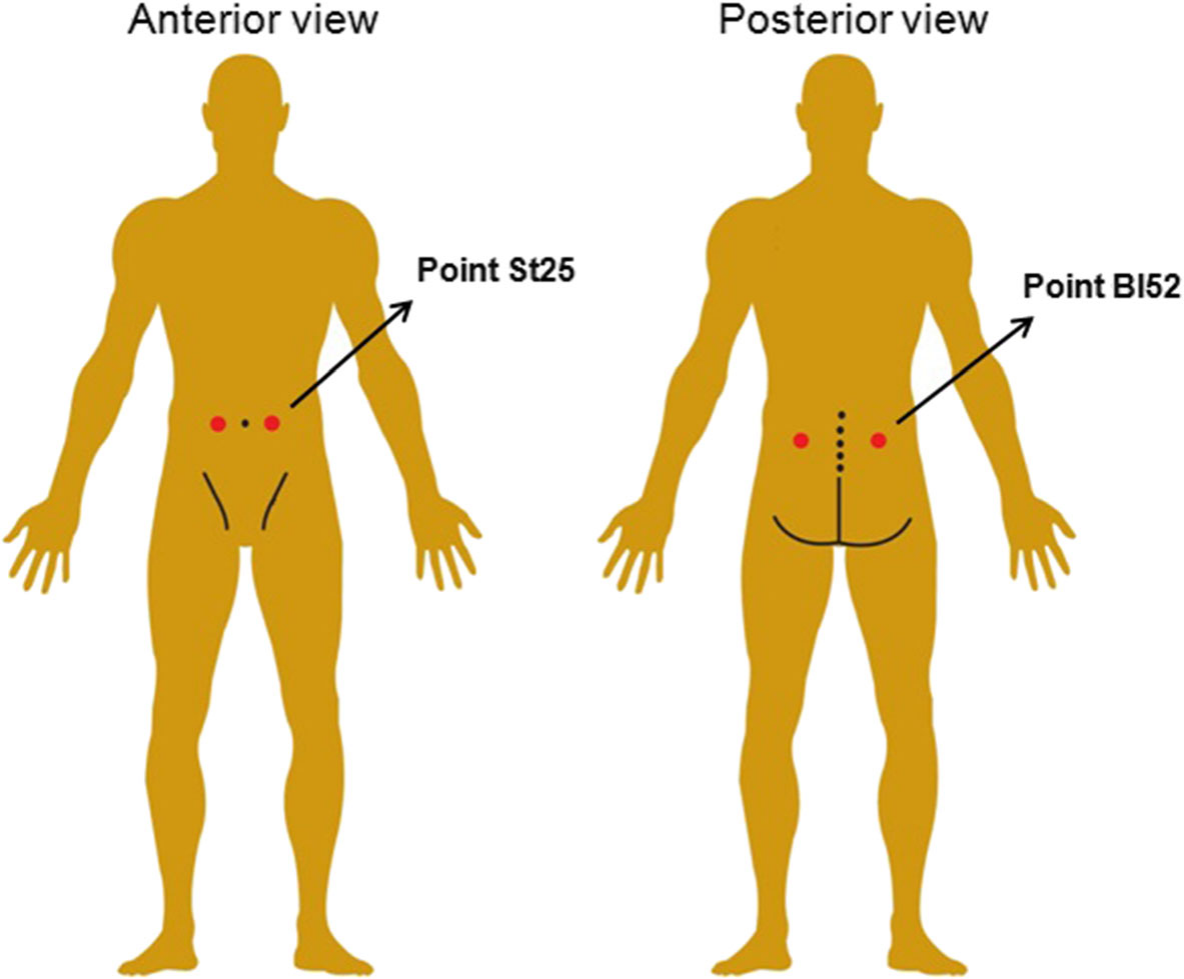
Acupuncture points: dorsal and ventral. Created by the author

Point ST25 is located on the upper abdomen region, 2 cun lateral to the center of umbilicus and BL52 point located in the lumbar region, at the same level as the inferior border of the spinous process of the second lumbar vertebra (L2), 3 cun lateral to the posterior median line [23]. These points were selected because they are part of the meridians widely considered to apply acupuncture in the clinical practice to treat back pain, intestinal and abdominal disorders, in addition to the use in both researches with animal [24, 25] and humans [26–31] (Fig. 1).

In the real acupuncture group, standard procedures were performed by expert acupuncturist, inserting the needle bilaterally to a depth of 10 mm [5] at the point corresponding to the group (BL52 or ST25) that was randomly selected, the needles remained in place for 30 min.

Group Park Sham (PS) used the device developed by Park et al. [16], in which the equipment is very similar to the real needle, but it has a guide tube inside another larger one, which is held together by adhesive perpendicular to the skin. When it is pressed, the smaller guide tube containing the needle inside, slides inside the larger tube; if the needle is conventional, it penetrates the skin, but if it is unconventional (blunt needle), just press the skin without penetrating, we use the second type of needle, because we want to use only the placebo park sham mode. Guide tube stuck by adhesive remained on the local of the puncture for 30 min, holding position similar to real needle after penetrate the skin.

The group Needle + foam used a self adhesive foam adhered to the skin, which was applied in order that the needle penetrated just the foam and not the skin, and then it remained vertically fixed for 30 min. In the group insertion and removal, real needles were used and inserted into acupuncture points, repeating the procedure such as in the real acupuncture group. However, they were removed immediately after insertion. Subjects remained 30 min without the needles inserted and at the end, the acupuncturist simulated the removal of needles with guide tube.

Groups that associated real acupuncture with some placebo techinique (Real + PS, real + Needle and foam, real + insertion and removal) performed the insertion of the real needle in a body side and one of the placebo techiniques on the contralateral side, so the same subjects could experience two procedures in their body, allowing a sensitivity comparison.

All subjects remained 30 min with needles or placebo device, applied by experienced and trained acupuncturist to perform all placebo methods, and the subjects were informed to not look at the local of needles insertion. After the performance of the technique, they all received a sticker where the application was made and were directed to the evaluation room to respond to the questionnaire applied by the researcher 1.

The volunteers were initially told that the aim of this study was to analyze the sensation promoted by different types of puncture, acupuncture techniques and only after participating of all stages of research, they had been told their real purpose. That was done to not influence the response of the questionnaire, which contained the following questions: 1) Was acupuncture a pleasant therapy for you?, 2) Do you felt some discomfort at the time of puncture?, 3) What was the intensity of discomfort on a scale of 0 until 10?, 4) Was the feeling located at the point stimulated?, 5) How long was this feeling?, 6) Do you think that your treatment was real or placebo?, just before the sixth question the researcher explained to volunteer the difference between a placebo and real therapy, after the explanation the subject could answer the question.

### Measurement of discomfort caused by cupuncture

The intensity of discomfort caused by puncture was investigated to evaluate the perception of this variable by the research subjects at the moment of puncture. For that, the 11-point numerical scale, ranging from 0 to 10, with zero indicating “no discomfort” and 10 indicating the “worst discomfort imaginable” was used, and the evaluator requested the subject to verbally classify its discomfort in this range [32].

### Study Procedures

**Fig. 2:**
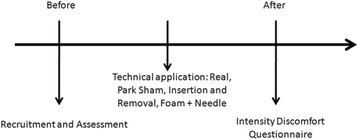
Timeline for assessment and treatment procedures of the study. Source: created by the author

Initially, a screening of healthy subjects that showed no discomfort in any region chosen for puncture was performed. Subjects who met the inclusion criteria were evaluated individually. In the evaluation sheet, demographic data such as age, height, weight, body mass index (BMI), educational level, marital status, occupation and questions about the use of alcohol, cigarettes and physical activity were recorded. Measurement of discomfort and the questionnaire was done only after the end of technique (Fig. 2).

### Statistical analysis

Research data were taken to an Excel data sheet for Windows 2007 and then to the Bioestat software, version 6.1, for the following analyzes: Shapiro-Wilk test to analyze the normality of the numerical variables, which indicated the need of the use of non-parametric tests for all variables. Kruskal Wallis test was used for analysis of body mass index, intensity of discomfort of the puncture and duration of the needling sensation. Post hoc test of Dunn was used for multiple comparisons when necessary. Chi square test was used for analysis of categorical variables. Data value of *p* < 0.05 were considered statistically significant.

## Results

### Volunteers

**Fig. 3:**
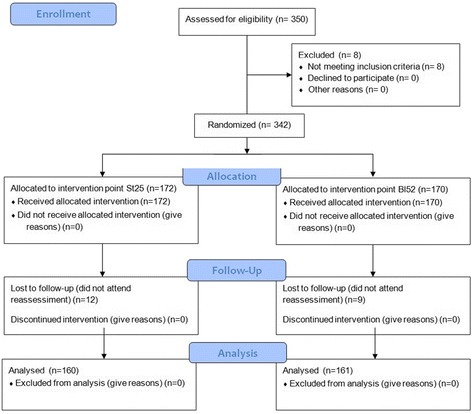
Representative subjects flow diagram for the steps of research

Three hundred and fifty subjects were recruited and evaluated to participate in. Of these, eight volunteers were excluded in the initial phase of data collection, because they fit into one or more exclusion criteria, 21 subjects did not appear to the reassessmet stage, and were therefore excluded, totaling 29 exclusions as described in Fig. 3. Thus the survey had 321 participants allocated in the 14 groups whose demographic and anthropometric characteristics are shown in Table 1.

### General perception of acupuncture

**Fig. 4:**
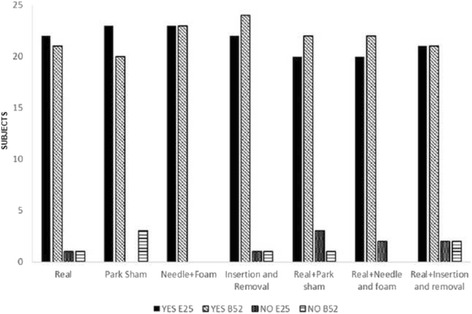
Presence of puncture sensation at the point ST25 or BL52. Values obtained from a questionnaire applied in the reassesment phase in all study groups. Values were presented as absolute frequency. Chi-Square test (*p* = 0.48)

There was no significant difference in the presence of prick sensation promoted by different placebo methods investigated (*p* > 0.05). The values of this response are presented in absolute frequency in Fig. 4 for all the groups that used the ST25 or BL52 points.

### Discomfort at the moment of puncture

**Fig. 5:**
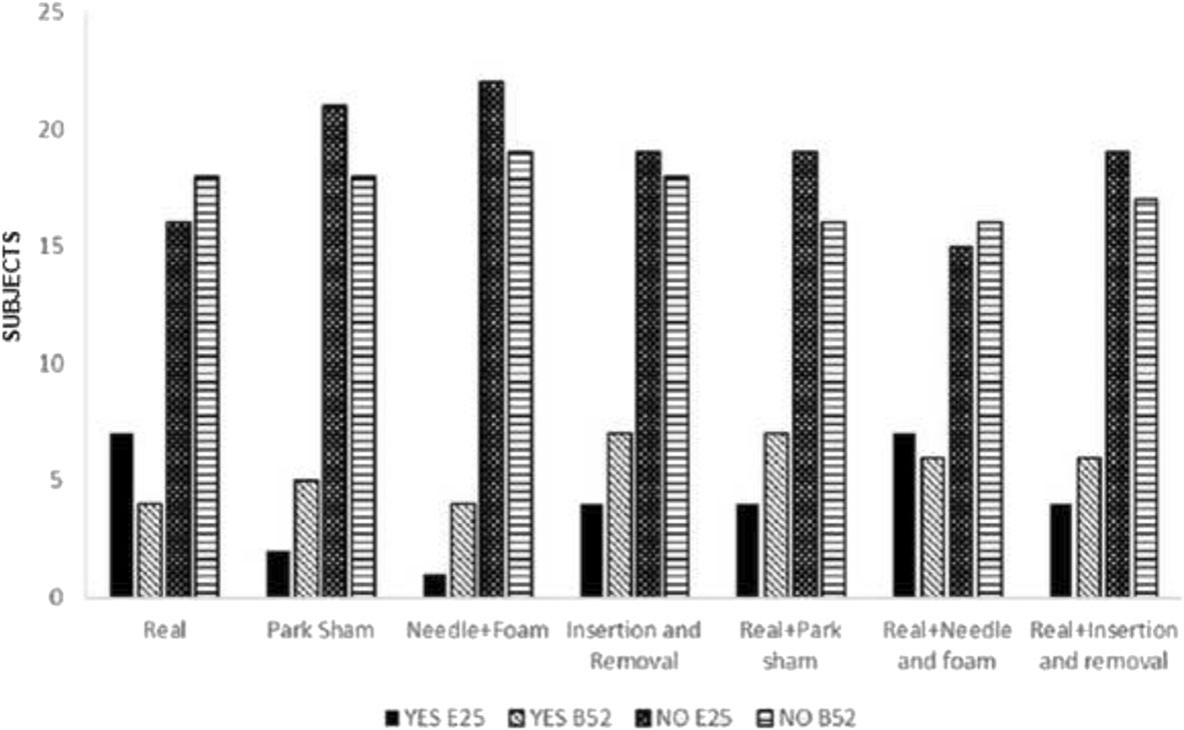
Presence of discomfort at the time of puncture at points ST25 and BL52. Values obtained from a questionnaire applied in the revaluation phase in all study groups. Values were presented as absolute frequency. Chi-Square Test (*p* = 0.5)

Presence of discomfort from the puncture did not differ significantly between study groups (*p* > 0.05), as shown in Fig. 5.

### Location of the feeling of puncture

Comparing the responses of all investigated groups there was no statistically significant difference in this variable.

**Fig. 6:**
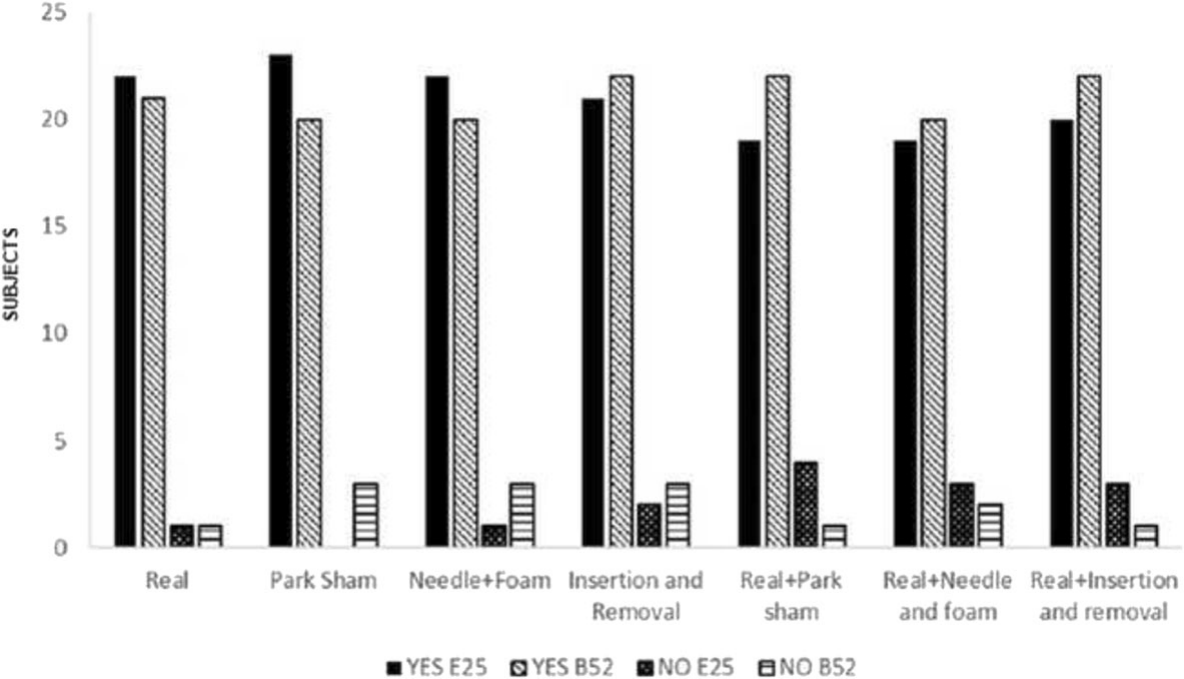
Sensation in the puncture points ST25 and BL52. Values obtained through a questionnaire applied in the reassessment in all study groups. Values were presented as absolute frequency. Chi-square test (*p* = 0.719)

Figure 6 represents both ST25 and BL52 points for the question posed at the time of reassessment that wondered whether the feeling percived by the volunteer at the moment of puncture was located or not in the stimulated point. Values are presented in absolute frequency.

### Intensity of discomfort

**Fig. 7:**
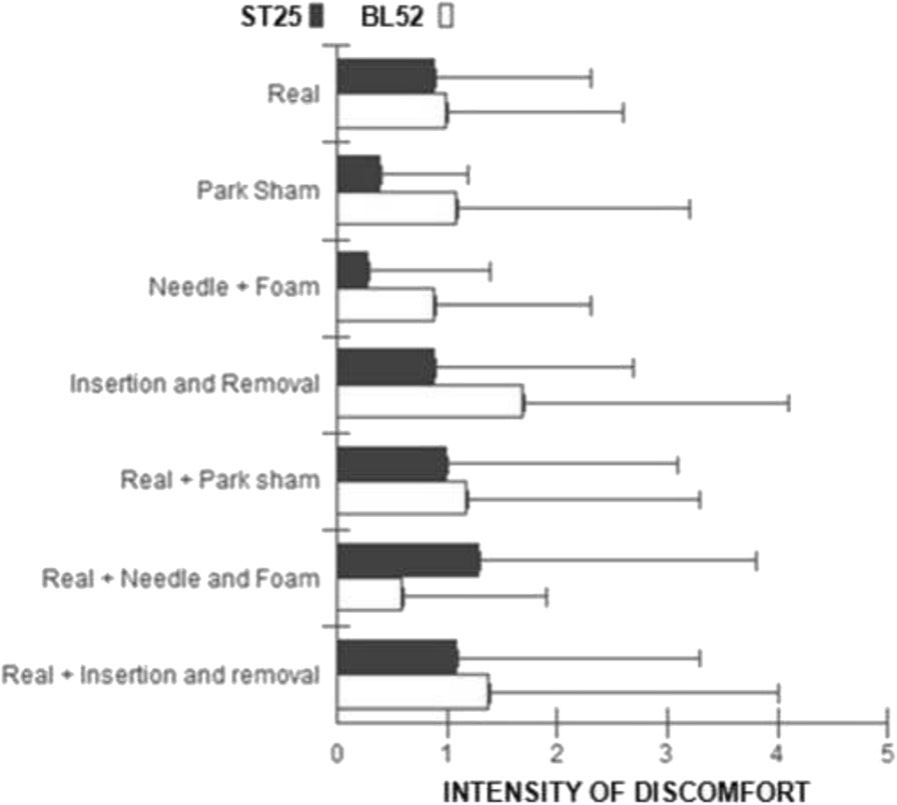
Intensity of discomfort caused by needling the points ST25 (*black*) and BL52 (*white*). Values measured using the numerical 11 points numerical scale in the reassessment phase in all groups. Values are presented as mean ± standard deviation. Kruskal Wallis test for the intergroup analysis (*p* = 0.768)

There was no significant statistical difference in the intensity of discomfort caused by the puncture between groups (*p* = 0.768) (Fig. 7).

### Duration of the puncture sensation

**Fig. 8:**
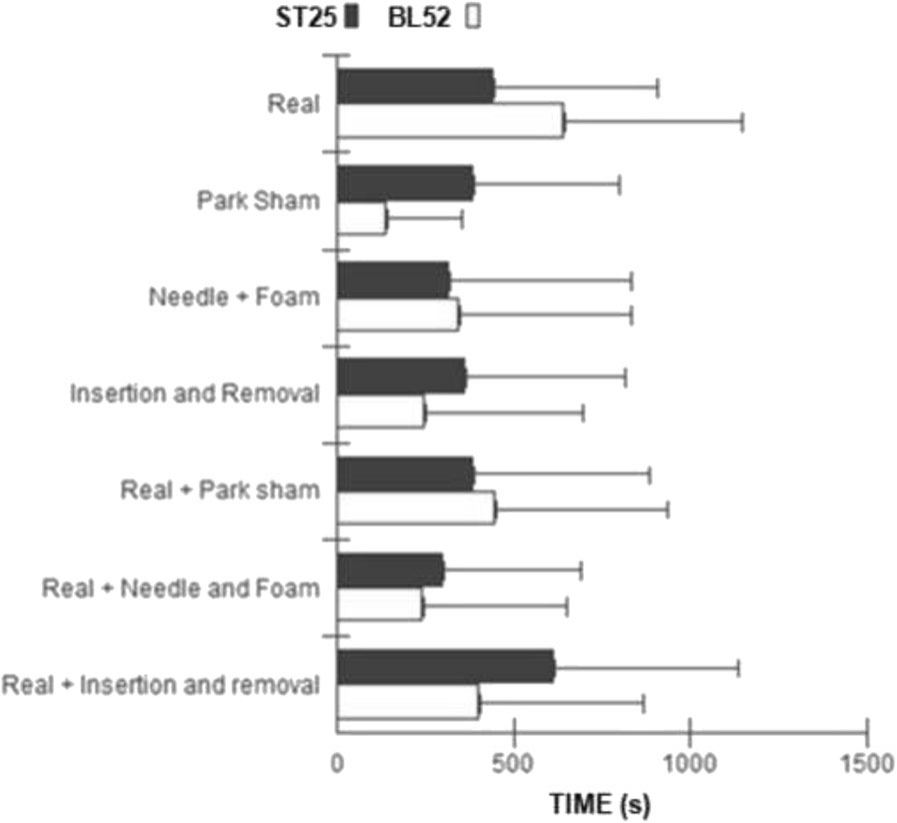
Duration of needling sensation (in seconds). Values reported by volunteers in the reassesment phase in all groups surveyed. The values are presented as mean ± standard deviation. Kruskal Wallis test (*p* <0.002) for inter-group analysis, adjusted by Dunn’s test (* *p* <0.002)

There was a significant higher value for the duration of sensation in Real BL52 point compared with Foam + Needle ST25, Park Sham BL52 and Real + Needle and foam on BL52 groups (*p* <0.05). Also, real group + insertion and removal on ST25 showed a significant higher needling sensation duration than the Real group + foam and needle in BL52 (*p* <0.05). The duration of the puncture sensation for ST25 and BL52 groups is shown in Fig. 8.

### Placebo vs. Real

**Fig. 9:**
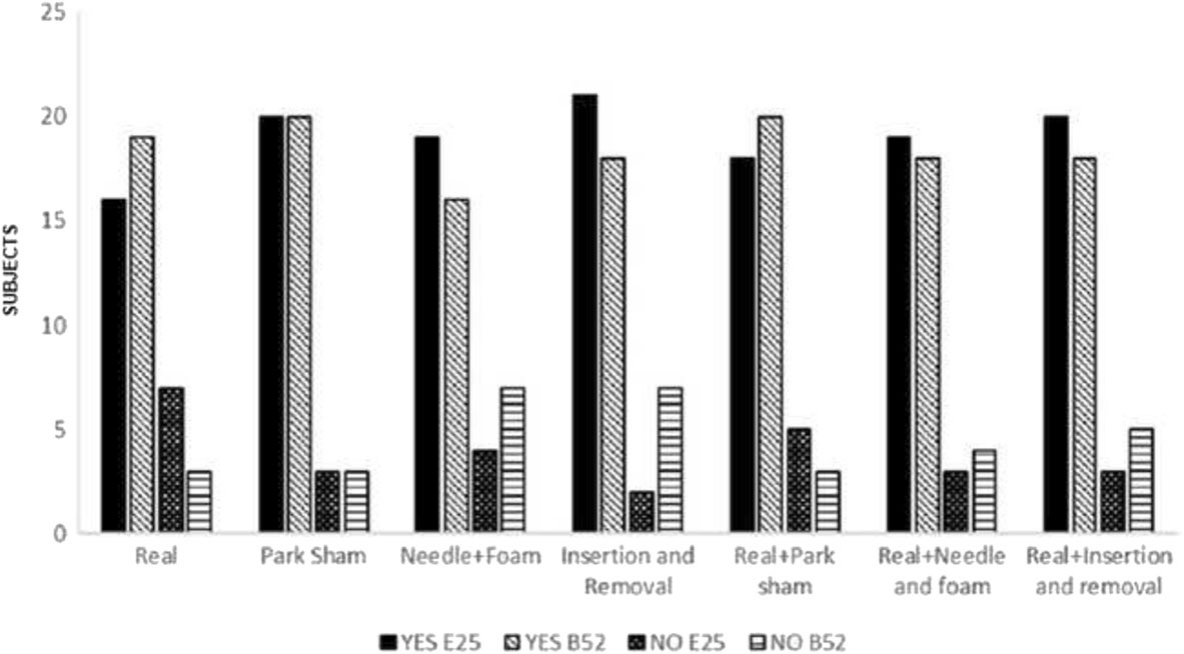
Response of the subject indicating whether they thought had received real or placebo procedure in point ST25 or BL52. Values obtained through questionnaire applied in the reassessment in all study groups. The values were presented as absolute frequency. ‘Teste Chi-Square (*p* = 0.677)

There was no significant difference between the groups when the subjects were asked whether they thought they were getting real acupuncture or placebo (Fig. 9 for all the groups).

## Discussion

The present study showed that all placebo acupuncture groups had similar results to the group that received real acupuncture for masking the patients, both at the ST25 BL52 points, suggesting that any of these placebo methods can be used in future research to simulate real acupuncture.

With regard to the occurrence of discomfort at the moment of puncture, no difference was found between subjects receiving placebo or real puncture, in both acupoints analyzed. This may not be considered as a key determinant aspect for the choice of placebo method, therefore besides analyzing the presence or absence of discomfort, respondents who reported discomfort were questioned about its intensity, which did not differ between groups.

The location for the needling sensation, also known as “Qi”, had its occurrence in puncture point in all groups investigated, and was not diffuse as reported in some studies [1, 33, 34], but when analyzing the duration of this sensation in each subject, the real group BL52 and Real group + insertion and removal ST25, showed a greater sensation period of needling than the others, corroborating with work. Similarly, Junnila [35] observed chronic pain patients also had a needling sensation for longer periods than 2 min.

In contrast, Park Sham BL52 group and Real group + needle and foam BL52 showed shorter duration for the needling sensation, and this sensation is one of the main events in individuals who receive real acupuncture therapy [34]. To our knowledge, this study is the first clinical trial to evaluate the efficacy of masking procedure of three methods of placebo acupuncture versus real acupuncture in healthy subjects.

The coping strategy of pain has an important role regarding to reduce pain intensity. Patient who undergoes to therapy and conveys confidence about its effectiveness, even if the subject feels to having control, or to being part of driving this therapy, it has been shown to be a catalyzing factor that results for pain relief [36], once that this one is one of the main reasons for seeking placebo acupuncture techniques that adeuately simulates as much as possible the feeling promoted by real acupuncture.

One of the main challenges faced by non-penetrating placebo acupuncture methods is to simulate the senation usually promoted by the puncture, since some studies reported that this sensation is directly related to the depth of needle penetration [33, 37]. According our findings, since the real group BL52 and real + foam and needle BL52 group, both of them using invasive procedures for stimulation, produced a greater time period for the puncture sensation than in noninvasive technique Park Sham BL52 group, which so the feeling sensation may be a determining factor to the volunteer research believes in the veracity of the technique.

The literature provides a large amount of placebo acupuncture techniques, such as Park Sham device, developed by Park et al., [16], which aims to serve as a masking to both patient and acupuncturist. In the research setting, for validation purposes, this device was firstly used in healthy subjects and then in patients who had suffered stroke, and, in both cases, the placebo device was not detected as placebo or not penetrating by the volunteers. These data confirm our findings, because the placebo acupuncture devices showed results similar to real acupuncture at both ST25 and BL52 points, when individuals were asked if they believed they had received real placebo treatment. Similar results were also found in other studies with healthy subjects [38–42].

Some placebo acupuncture devices that intended to mask both acupuncturist and patient were not effective to do it as acupuncturists found that it was a fake device, while the volunteers believed it was real acupuncture [43, 44]. Other studies have shown that placebo acupuncture technique is not effective to mask even the patient [1, 45]. Mechanical changes were measured by using computerized system, and showed that, at the moment of insertion and removal of needles, professional modifies the forces of application and removal when using placebo methods compared to real acupuncture [46], which is always a possibility of perception for the patient who has previous experience with acupuncture, to identify the difference on the technique, which was not observed in our study because we measured a high credibility index.

In our study, we had groups that used real acupuncture in a body area and a placebo method in another one in the same subject, in order to simulate the occurrence of individuals who had previous experience with acupuncture. Interestingly, the result for masking the participants was similar to the groups receiving real acupuncture or placebo device. In a study with eight experienced acupuncturists in order to develop a regulation for usig a method of non-invasive sham acupuncture, it became clear that to maintain an appropriate standard is required frequent contact between all professionals who will apply the placebo technique [47].

Studies with real acupuncture and sham acupuncture showed similar therapeutic results to increase muscle strength in the quadriceps [38], treatment of low back pain [8] and for pain relief in knee [48], suggesting that the therapeutic results of this study has a strong relationship with the placebo effect. However, studies in which acupuncture was applied in patients with muscle pain in the upper trapezius [49] or with nonspecific low back pain [21] pointed out that the real acupuncture therapy was more effective than placebo acupuncture for pain relief.

Our findings indicated a low level of discomfort at the moment of puncture in subjects treated with all the methods investigated in both acupuncture points, however, this intensity was not different between groups; this suggests that the intensity of discomfort can not be considered a determining factor for the choice of placebo method to be used in clinical trials investigating the effects of acupuncture. These data corroborate findings by Hübscher et al. [38] that evaluated the effect of acupuncture and placebo group with no intervention in muscle soreness, noting that no difference in the intensity of discomfort was observed in the first 48 h between study groups. Whereas after 72 h, only the acupuncture group showed a significant reduction in the intensity of discomfort.

Subjects were asked if acupuncture was pleasant or not. Most of them in all groups said that acupuncture had been nice and no differences were found between the methods, showing once again that all placebo and real acupuncture techniques were similar to each other for the pleasantness factor, which should be regarded as a decisive factor for the choice of the placebo method to be used in the clinical research setting, whether in the abdominal point ST25 or lumbar point BL52.

Our findings contribute to modern science that seeks to avoid erroneous conclusions in no placebo-controlled studies, which provides differences in results between the groups investigated, regardless of the odds and potential that the belief of healing provides these improvements [50]. A systematic review with 250 random clinical trials demonstrated superior therapeutic effect, around 17%, for the groups that did not use masking in comparison to the groups who performed [51].

Our clinical trial occurred with random distribution and volunteer was unaware about the acupuncture technique that would be received, researchers responsible for the assessment also had no knowledge about allocation, and were trained to conduct the survey question about the subjects’ belief if they received placebo or real admnisration of needle, characterizing it as a double-blinded study. Studies that mask the volunteers as the evaluators have fundamental importance, because the person who is aware that a real treatment method was not performed will be less prone to follow the protocol of the test, search for additional treatment and making the data less reliable, as well as blind investigators are less likely to transfer expectations to volunteers ensuring more impartial data [52, 53].

## Conclusion

The results allow to state that all the placebo groups were similarly effective to promote appropriate masking of the study subjectsand therefore all of them are reliable alternatives to characterize a placebo-controlled group in the research setting using acupuncture at either abdominal or lumbar points. All groups showed low intensity of sensory discomfort, which was similar between the methods studied. Furthermore, the puncture sensation was reported as located by most individuals in all groups for the ST25 and BL52 points. The groups that had a higher maintenance puncture sensation time were those stimulated with real acupuncture at acupoint BL52 and real acupuncture + insertion and removal in acupoint ST25. Most individuals of all groups have reported that the acupuncture treatment was a pleasant therapy received.

## Abbreviations

Bl 52: Bladder 52
St 25: Stomach 25
